# Surgical residents' perceptions of the operating theatre educational environment at St. Paul's Hospital Millennium Medical College: A cross-sectional survey

**DOI:** 10.1016/j.sopen.2023.12.011

**Published:** 2023-12-31

**Authors:** Goytom Knfe, Henok Teshome, Maru Gama, Engida Abebe, Mulugeta Kassahun, Berhanetsehay Tekelwold

**Affiliations:** Department of Surgery, St. Paul's Hospital Millennium Medical College, Addis Ababa, Ethiopia

**Keywords:** Medical education, Learning environment, Operation theatre, Surgical residents, Perception

## Abstract

**Background:**

The educational environment refers to the “climate” that influences all aspects of learning in an educational context and the experience in the operating room is particularly crucial in surgical residents learning. Hence, this study aimed to assess surgical residents' perceptions of the operating theatre educational environment and associated factors in the surgical department at St. Paul's Hospital.

**Methods:**

This cross-sectional study was conducted in March 2022 among surgical residents at St. Paul's Hospital Millennium Medical College to assess their perceptions of the operating room educational environment using the OREEM questionnaire. Descriptive statistics (mean, median, SD) were used to summarize demographic data and OREEM scores. The student *t*-test and one-way analysis of variance (ANOVA) testing followed by posthoc tests were used for comparison of quantitative data, with *p*-values < 0.05 considered significant.

**Results:**

Of the participants, 103 (79.8%) were male and 26 (20.2%) were female with a mean age of 28 years. The overall mean score was 69% with subscale scores for teaching and training at 47.9/65.0 (73.7%), learning opportunities at 34.5/55.0 (62.7%), the atmosphere at 28.9/40.00 (72.4%) and workload/supervision/ support at 27.5/40.0 (68.7%). Male and female residents differed significantly in perceptions of “atmosphere” (t_127_ = 3.35, *p* < 0.001) and in junior versus senior residents' perceptions of the “learning opportunities” and “atmosphere” at *p*-values of 0.023 and 0.028 respectively. However, age, marital status, and specific surgical training programs did not have a significant effect on the scores.

**Conclusion:**

Overall, residents had positive perceptions of their training and teaching, learning opportunities, the atmosphere in the operation theatre, and the supervision they received in the operation theatre. The operating room's “teaching and training” component received the highest score, while the operating room's “learning opportunities” component received the lowest. This indicates the importance of establishing a positive learning environment with sufficient “hands-on” experience, especially during emergencies. In addition, preoperative planning, case discussions, and feedback after the surgery should be routine.

## Introduction

The educational environment is an integral part of the educational program and [1] has been defined as how students or faculty comprehend the medical climate underlying all educational aspects in an academic setting [2]. Surgical residency programs, characterized by an apprenticeship model, necessitate extensive training in the operating room, where technical skills and knowledge are honed through supervised exposure and didactic sessions [[3], [4], [5]]. However, the translation of these educational tenets into effective practice remains a formidable challenge for trainers, with the learning environment subject to the complexities posed by individuals, case intricacies, and potential distractions [3].

The optimization of the educational experience within the operating theatre is crucial for surgical residents who invest over 10,000 h in clinical training, making it the cornerstone of their professional development [3]. Mastery of surgical skills is intrinsically tied to ample practice and sustained mentorship, positioning operative experience as a key predictor of satisfaction with surgical training [6,7]. Despite its recognized significance, studies reveal that a substantial percentage of surgical residents express dissatisfaction with their operative experiences, ranging from 65% to 85% [8,9]. Moreover, a systematic review indicates that the reduction in duty hours has led to a decline in trainees' operative experience, impacting their performance in certification exams [10].

The assessment of the educational environment in the operating room is essential not only for gauging resident satisfaction but also for ensuring the quality of surgical residency programs [[11], [12], [13]]. This evaluation extends beyond individual perceptions, encompassing factors such as time management, resource availability, roles, patient safety, and sterility, which collectively contribute to the overall educational experiences. Concerns about these elements create tension within the surgical team, underscoring the need for a holistic understanding of the clinical learning environment [14].

While some studies report satisfactory overall mean scores in the assessment of the educational environment, disparities exist across various subscales, such as workload/supervision/support, indicating areas for potential improvement. Disparities in perceptions of the clinical learning environment within surgical residency programs have been demonstrated across gender, program type, year of residency, and geographical differences [[13], [14], [15], [16], [17]]. The implications of these perceptions extend beyond personal satisfaction, influencing the quality of patient care, learning outcomes, and the potential for burnout and stress among both learners and educators [13].

Divergent perceptions of the OR learning environment in surgical residency programs have emerged from international studies. In Nigeria, an overall mean score of 67.5% unveiled nuanced differences, particularly in the operating theatre atmosphere (79.2%) and the challenging workload/supervision/support subscale (48.3%), compounded by gender-based variations [14]. Similarly, general surgery residents consistently reported lower satisfaction than their peers in other specialties [13]. Concurrently, Saudi Arabian urology residents expressed dissatisfaction, citing the pivotal role of a surgeon's teaching approach [17]. Meanwhile, in Ethiopia, the surge in surgical resident enrollment has strained resources, leading to reduced operating hours and concerns about personalized faculty engagement, highlighting the need for a comprehensive understanding of diverse factors influencing the educational landscape in surgical training programs globally [10,18].

Despite the high unmet need for surgeons, inadequate operating room teaching, and the fact that the operating room is an expensive teaching venue, few studies have been conducted on this topic in Ethiopia. A study from Addis Ababa University highlighted the alarming discrepancy in the perception of surgical residents and faculty regarding intraoperative teaching [6] but did not capture the perception of surgical residents towards the OR educational environment and the associated factors. Hence, the present study aimed to build upon the evidence identified in previous studies by evaluating the surgical trainee's objective perspective and the factors associated with the current operating room educational environment in the surgical department at St. Paul's Hospital Millennium Medical College (SPHMMC) using an appropriate instrument. The operation room educational environment measure (OREEM), a validated tool comprising 40 Likert-type items divided into four subscales, is widely used to assess the educational environment in the operating theatre [13,15].

## Objectives

### General objectives

The purpose of this study was to assess surgical postgraduate residents' perceptions of their learning environment in operating rooms and associated factors at SPHMMC, Addis Ababa, Ethiopia in 2022 G.C.

### Specific objectives

1. Assess the perception of surgical residents about the operating theatre educational environment at SPHMMC
2. Identify factors that influence the operating theatre educational experiences of surgical residents at SPHMMC

## Methods

### Study setting and period

An analytical cross-sectional study was conducted from July 1 to August 30, 2022, at St. Paul's Hospital Millennium Medical College (SPHMMC), the second-largest multi-specialty tertiary care teaching hospital in Ethiopia, is located in Addis Ababa. In 2013, a surgical residency-training program was started with seven residents who graduated from five batches of general surgeons. Currently, >*100* residents are enrolled in specialties and subspecialty programs. The department also provides fellowship programs for endocrine, breast, hepatobiliary, and pancreatic surgery [19].

### Study subjects

Convenience sampling was used to select participants. The sample size was calculated based on a prior presumption that the level of perception of the learning environment was 50% since there has not been a published study in the past in an Ethiopian surgical residency program with the desired precision of d = 0.05. The sample size was determined using the single-population proportion formula and adding a nonresponse rate of 10%. The final sample size calculated for this study was 94 residents, and given that the sample size is comparable to the total number of residents, all the surgery residents (postgraduate years 1–4) who had been actively working in the hospital for at least six months were included in the study. Residents on sick leave, maternity leave, and currently on detachment sites were excluded from the study.

### Study instrument and procedure

Within the medical environment, there are several instruments developed such as the Dundee Ready Educational Environment Measure (DREEM), Postgraduate Hospital Educational Environment Measure (PHEEM), Anesthetic Theatre Educational Environment Measure (ATEEM), and Operation Theatre Educational Environment Measure (OREEM), to name a few. The OREEM scale stands out as the most extensively used specific measure for evaluating the OT educational environment across all of the reviewed studies [13,16,20]. OREEM was administered to surgery residents at St. Paul's Hospital, Millennium Medical College, using a paper-based questionnaire. Residents were asked to respond to a set of 40 statements related to the operating room educational environment with the use of a 5-point Likert scale, with possible responses ranging from “strongly agree,” “agree,” “undecided,” “disagree,” and “strongly disagree.” The inventory also requested information about the age, gender, marital status, and training program of the resident, the level of training by postgraduate year, and the global satisfaction score out of 100.

The 40 items of the questionnaire were divided into four major subscales. Inventory items 1 through 13 address the residents' perceptions of the preceptor or “surgical attending” in teaching and training; items 14 through 24 address the residents' perceptions of learning opportunities; items 25 through 32 cover the residents' perceptions of the atmosphere in the operating room; and items 33 through 40 relate to the residents' perceptions of the workload, supervision, and support. The residents completed the inventory on different dates, and thus, responses were in reference to different consultants and operating room experiences across the totality of their training.

The minimum score was 40 and the possible maximum score was 200. A score of at least 120 out of 200 was considered favorable. A value above 120 indicates a more satisfactory perception of the educational environment as indicated by most studies conducted using this parameter [7,[21], [22], [23]]. A few items with negative responses (*8, 14, 19, 22, 23, 26, 27, 28, 30, 31, 33, 34, 35, 36, 37, 28, and 40*) were reverse-coded to keep the score in the positive direction.

### Data processing and analysis

Data processing began by checking the data gathered for accuracy and completeness. Each completed questionnaire was assigned a unique code and entered into a computer using *epi*. Info version 3.5.1 and exported to SPSS version 26.00 for further analysis. A summary of the data was presented using frequency distributions, graphs, and plots. Continuous variables are described as mean and standard deviation and categorical variables as frequencies and percentages. Student's *t*-test and one-way ANOVA were used as methods of inferential statistics to assess any significant differences based on gender and marital status, year of training, and training program. Spearman's correlation was also calculated to find any association between the various subscales of the questionnaire and the global satisfaction score. The confidence interval was set to 95%, with a 5% margin of error.

### Data quality control

A pilot study was conducted on 5% of randomly selected residents to ascertain whether the questions and instructions were sufficiently understood or required revision and additional instructions. Following these modifications, the questionnaire was distributed to the study population. Questions raised by the residents regarding confusing or unclear statements were cleared by the principal investigator. Additionally, all the collected data were reviewed and checked for completeness upon submission.

### Ethical clearance

Clearance was obtained from the Institutional Research and Ethics Review Board (IRB) of SPHMMC. The study objectives and anticipated potential risks and benefits were explained to the residents. Participants were assured that their data and information would be kept confidential and that they would not be shared outside the concerned bodies. To protect the confidentiality of information, names or other personal identifiers were not included in the questionnaires.

## Results

### Sociodemographic characteristics

Of the respondents, 103 (79.8%) were male and 26 (20.2%) were female, with a mean age of 28.5 + 2 Years. The respondents were distributed across the levels of training from postgraduate years (PGY) 1 to 5. (PGY-I residents comprised 42(32.6%), PGY-2 residents, 28(21.7%); PGY-3, 38(29.5%); PGY-4, 13(10.1%); and PGY-5, 8(6.2%). Surgery residents from different departments were included in the study, and the majority of participants 53(41.1%) belonged to the general surgery department, followed by orthopedic surgery (25(19.4%)), urology (9.3%), and neurosurgery (9.3%). Most of the residents were single (67.4%). The residents were located at two training sites in Addis Ababa, with the two main teaching hospitals being AaBET Hospital (plastic surgery, orthopedics, and neurosurgery; 25.1% of residents), and the rest at St. Paul's Hospital Millennium Medical College (Table 1).

**Table 1:** Characteristics of surgical residents at St. Paul Hospital Millennium Medical College, teaching hospitals located in AA, Ethiopia in 2022 G.C. (*n* = 129).

Characteristics		No. of participants	
		No.	%
Sex	Male	103	79.8
	Female	26	20.2
Postgraduate	I	42	32.6
	II	28	21.7
	III	38	29.5
	IV	13	10.1
	V	8	6.2
Marital status	Married	36	27.9
	Single	87	67.4
	Engaged	5	3.9
	Other	1	0.8
Training Program	General Surgery	53	41.1
	Pediatric surgery	5	3.9
	Urology surgery	12	9.3
	Neurosurgery	12	9.3
	Plastic surgery	7	5.4
	Orthopedics	25	19.4
	ENT	9	7.0
	Maxillofacial	6	4.7

### OREEM Scale and subscale scores

**Fig. 1:**
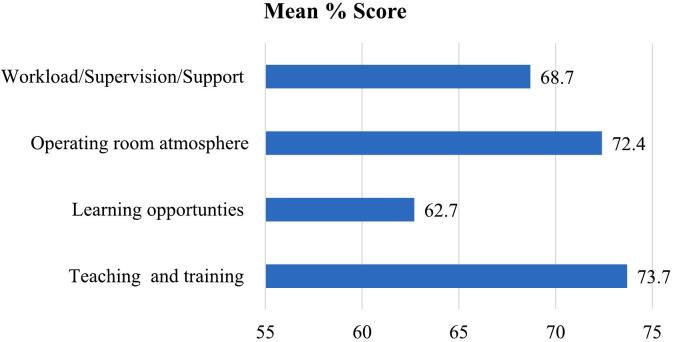
Mean percentage total score and percentage score for each subscale among surgical residents at St. Paul Hospital Millennium Medical College, teaching hospitals located in AA, Ethiopia in 2022 G.C. (*n* = 129).

The mean total score was 138.8/200 (69%). The mean scores on the subscales were as follows: teaching and training 47.9/65.0 (73.7%); learning opportunities 34.5/55.0 (62.7%); atmosphere 28.9/40.00 (72.4%); and workload/supervision/ support 27.5/40.0 (68.7%). Analysis of subscale scores showed that the operating room “teaching and training” subscale had the highest score, with a score of 73.7%. The lowest scoring subscale was ‘learning opportunities’ (62.7%) (Fig. 1).

The three highest scoring items were no. 6 “My preceptor's surgical skills were very good. (4.4+0.7)”, no. 31 “I (do not) feel discriminated against in the operating room because of my race. (4.3+1.04)” and no. 30 “I (do not) feel discriminated in the operating room because of my sex. (4.3+1.07)”. Moreover, the 7 items with “satisfactory” mean scores (mean item score > 4) were, “My attending has a pleasant personality,” “I get along well with my attending,” and “I understand what my attending is trying to teach me,” “My attending's surgical skills are very good,” “I feel discriminated against in the operating room because of my sex,” “I feel discriminated against in the operating room because of my race,” and “I am(not) asked to perform operations alone that I do not feel competent at performing.”

The remaining questionnaire items were found to be less than satisfactory (mean item score <4), indicating the need for further investigation and improvement. The three lowest-ranked items were statements no. 27 “The nursing staff dislike it when I operate as the operation takes longer. (2.4+1.0)”, no. 19 “More senior residents or consultants take my opportunities to operate. (2.68+1.08)” And no. 20 “The number of emergency procedures was sufficient for me to gain the correct operative experience. (2.8+1.25)”. These items should be prioritized in future program evaluations (Table 2, Table 3, Table 4, Table 5).

**Table 2:** Responses to the OREEM survey Likert questions: Subscale teaching and training among surgical residents at St. Paul Hospital Millennium Medical College, teaching hospitals located in AA, Ethiopia in 2022 G.C. (*n* = 129).

Teaching and training	Mean score	+SD
My consultant has a pleasant personality.	4	0.8
I get on well with my consultant.	4	0.7
My consultant is enthusiastic about teaching.	3.94	0.8
My consultant has a genuine interest in my progress.	3.78	0.9
I understand what my consultant is trying to teach me.	4.17	0.8
My consultant's surgical skills are very good.	4.42	0.7
My consultant gives me time to practice my surgical skills in theatre.	3.74	1.0
My consultant immediately takes the instruments away when I do not perform well.	3.04	1.1
Before the operation, my consultant discusses the surgical technique planned.	3.32	1.1
Before the operation, my consultant discusses what part of the procedure, I will perform.	2.99	1.1
My consultant expects my surgical skills to be as good as his/her.	3.12	1.2
My consultant gives me feedback on my performance.	3.51	1.0
My consultant's criticism is constructive.	3.81	0.9

**Table 3:** Responses to the OREEM survey questions: Subscale learning opportunities among surgical residents at St. Paul Hospital Millennium Medical College, teaching hospitals located in AA, Ethiopia in 2022 G.C. (*n* = 129).

Learning opportunities	Mean score	+ SD
The type of operations performed on this rotation is too complex for my level.	3.39	1.1
The elective operating list has the right case mix to suit my training.	3.55	1.1
There are too few cases on the elective list to give me the opportunity to operate.	3.17	1.1
I get enough opportunities to assist.	3.18	1.1
There are enough operating theatre sessions per week for me to gain the appropriate experience.	2.89	1.1
More senior residents or consultants take my opportunities to operate.	2.68	1.1
The number of emergency procedures is sufficient for me to gain the right operative experience.	2.79	1.2
The variety of emergency cases gives me the appropriate exposure	3.04	1.2
My consultant is in too much of a rush during emergency cases to let me operate.	3.41	0.9
I miss out on the operative experience because of restrictions on working hours.	3.30	1.1
I have the opportunity to develop the skills required at my stage.	3.08	1.1

**Table 4:** Responses to the OREEM survey questions: Subscale operating theatre atmosphere among surgical residents at St. Paul Hospital Millennium Medical College, teaching hospitals located in AA, Ethiopia in 2022 G.C. (n = 129).

Operating theatre atmosphere	Mean score	+ SD
The atmosphere in the operating theatre is pleasant.	3.66	1.0
In the operating theatre, I don't like being corrected in front of medical students, nurses, and residents.	3.96	0.9
The nursing staff dislikes it when I operate as the operation takes longer.	2.36	1.0
The anesthetists put pressure on my consultant to operate himself to reduce anesthetist time.	2.74	1.1
The staff in the operating theatre is friendly.	3.66	0.9
I feel discriminated against in theatre because of my sex.	4.26	1.1
I feel discriminated against in theatre because of my race.	4.31	1.0
I feel part of a team in theatre.	3.98	0.9

**Table 5:** Responses to the OREEM survey questions: Subscale workload/supervision/support among surgical residents at St. Paul Hospital Millennium Medical College, teaching hospitals located in AA, Ethiopia in 2022 G.C. (n = 129)*.*

Subscale workload/supervision/support	Mean score	+ SD
I am too busy doing other work to go to the theatre.	3.26	1.1
I am often too tired to get the most out of theatre teaching.	3.19	1.0
I am so stressed in the operating theatre that I do not learn as much as I should.	3.26	1.1
I am asked to perform operations alone that I do not feel competent at.	4.10	0.9
When I am in the theatre, there is nobody to cover the ward.	3.72	1.1
I get called during operations.	3.25	1.2
The level of supervision in theatre is adequate for my level.	3.54	1.0
The operative cases are too long.	3.16	1.2

### Validity and Reliability analysis

The reliability analysis was performed using Cronbach's alpha coefficient which was computed for the overall questionnaire and for each of the four subscales to measure the internal consistency of the Questionnaire. The Cronbach's alpha values were interpreted according to Richardson's suggestion. The Cronbach's alpha for the overall questionnaire was 0.865. The following were the Cronbach's alpha values for the factors indicated: “teaching and training” subscale-0.868; “supervision/ workload/support” subscale-0.770; “learning opportunities” subscale −0.684; “atmosphere’” atmosphere subscale −0.723. All Cronbach's alpha coefficients were considered to be within the acceptable to good range, except for learning opportunities. However, when our data was analyzed to exclude each question in turn, using the “alpha if item deleted”, no significant improvement was seen with the removal of any of the 40 questions. Spearman rho correlation was calculated for the overall score as well as subscales and found a strong positive correlation among all subscale scores and overall score (p-value < 0.05 with r-value ranging from 0.56 to 0.8).

### Factors associated with OREEM scale score

Comparisons were made between gender, junior and senior level residents, marital status, and different departments. No significant difference was identified regarding the OT teaching perception according to marital status and the different departments in the one-way ANOVA analysis. In the two-sample t-test analysis for gender, a statistically significant difference (t_127_ = 3.35, p < 0.001) was shown in the “atmosphere” subscale score between females (26.3/40 or 65.7%) and males (29.6/40 or 74%). The average “atmosphere” subscale score for females was lower than the mean subscale score for males by 3.35. This was corroborated by item analysis that revealed responses to items 29, 30, and 31, which comprise part of the “atmosphere” subscale, differed significantly between the two sexes. In addition, items 2 and 25 also were noted to contribute to the difference in overall scores.

Other items also were rated less favorably by female respondents including statement no. 6 “My consultant's surgical skills are very good. (t_127_ = 2.95, *p* < 0.004)”, and no. 9 “Before the operation my consultant discusses what part of the procedure I will perform. (t_127_ = 2.7, *p* < 0.008)”, whereas no. 8 “My consultant immediately takes the instruments away when I do not perform well. (t_127_ = −2.07, *p* < 0.040)” was rated more favorably by females. However, there was no statistically significant difference in the overall total score of the OREEM measure between males and females.

The level of training affected the perception of the residents about the OR learning environment mainly regarding “learning opportunities” and “atmosphere.” The mean score for “learning opportunities” for junior and senior residents was 33.4 + 6.4and 35.8 + 5.4, respectively (*p* < 0.023), while the mean score for “atmosphere” was 28.1 + 5.04 for juniors and 29.9 + 4.07 for seniors (*p* < 0.028). Six out of the 40 OREEM items (no. 3,14,17,20,26, and 17) were statistically different between junior and senior residents (*p* < 0.05). The overall OREEM mean score for junior and senior residents, however, was comparable with no significant difference (Table 6).

**Table 6:** Association between the residents' background information and Operating Room Educational Environment Measure (OREEM) scores among surgical residents at St. Paul Hospital Millennium Medical College, teaching hospitals located in AA, Ethiopia in 2022 G.C.

Characteristics		Operating Room Educational Environment Measure^a^				
		Trainer & Training	Learning Opportunities	Atmosphere in the OR	Supervision, Workload and Support	Overall
Gender	Female	3.56+ 0.66	3.16 + 0.51	3.28 + 0.60	3.52 + 0.76	3.39 + 0.38
	Male	3.7+ 0.57	3.12 + 0.56	3.70 + 0.56	3.41 + 0.67	3.49 + 0.42
	**p-value**⁎	0.238	0.788	0.001*	0.46	0.27
Level of training	Junior residents	3.7 + 0.57	3.03 + 0.58	3.5 + 0.63	3.49 + 0.64	3.45 + 0.44
	Senior residents	3.6+0.61	3.26 + 0.48	3.74 + 0.51	3.36 + 0.74	3.5 + 0.39
	**p-value**⁎	0.399	0.023*	0.028*	0.309	0.478
Department	General surgery	3.66+0.54	2.85+0.48	3.57+0.58	*3.63+0.55*	3.41+0.40
	Pediatric surgery	3.83+0.85	3.58+0.51	3.83+0.53	3.48+0.38	3.69+0.56
	Urology	3.75+0.44	2.92+0.39	3.94+0.48	*3.59*+*0.48*	3.53+0.32
	Plastic surgery	3.27+0.75	3.22+0.27	3.39+0.61	3.01+0.67	3.23+0.39
	Orthopedics	4.04+0.43	3.47+0.51	3.74+0.53	*3.48*+*0.83*	*3.71*+*0.41*
	Neurosurgery	3.39+0.56	3.31+0.53	3.53+0.40	***2.73***+*0**.73***	*3.26+0.39*
	ENT	3.31+0.87	3.28+0.68	3.91+0.63	*3.61+0.38*	3.48+0.51
	Maxillofacial	3.77+0.23	3.64+0.36	2.75+0.55	2.81+0.89	3.34+0.07
Department	**p-value**⁎	0.186	0.119	0.001	0.052	0.169

Bold and italic ones are statistically significant.

a Values reported are mean and standard deviation (SD).

⁎ Student t-test was used for “Gender” and “Level of training,” while a 1-way ANOVA test was used for “department.”.

### Free text responses and comments

The responses to the following three open-ended questions were collated and analyzed below.

1. Other comments on OT teaching?
2. What are the strengths of OT teaching at your department?
3. In what areas could operation theatre teaching be improved?

Seventy-eight respondents (60%) contributed at least one free-text comment. The comments mostly focused on the inadequate quantity and variety of procedures especially emergency procedures forwarded by 52(40%) of the surgical residents, and they outlined some causes for this. First, the cases referred to the college for surgery are more challenging and require sophisticated care because it is a referral hospital. Second, given the high number of residents recruited each year, particularly in the department of general surgery, the ratio of residents to cases operated on is noticeably high. Additionally, this mismatch contributes to the fact that residents will only have a few numbers of emergency duty hours and senior residents will scrub in many procedures, limiting junior residents' exposure to those cases.

Increasing the number of detachments to locations where there is a higher chance of exposure to a large number and variety of emergency cases was urged by 30(24%) of the surgical residents. The overall OT teaching and learning endeavor in the separate departments working on patients requiring assistance from additional essential devices is constrained by the frequent unavailability of high-tech equipment like the c-arms, multiple eyepieces operating microscopes, and endourological instruments. The comments listed as additional points include some of the following.

• “The senior staff OR nurses, and anesthesia team should understand that no one is born with knowledge and skills, and the junior residents are here to learn.”
• “Allowing the resident to practice under supervision should be the primary step to developing skills and confidence rather than doing the procedure and making them assistant most of the time!”
• “There are many things to be done by junior residents which limit teaching and exposure in OR team.”
• “Junior residents are called to bring blood or something else and hence missing the whole or some parts of the procedures. It doesn't encourage junior residents, as they are treated as an intern.”
• “The OR materials are of poor quality and quantity so I waste most of the time searching for instruments that should be available in the OR……instruments like arm support, leg support, rubber sheet, pillow, OR light, suctioning machine.”

Numerous respondents (38(30%)) emphasized the importance of preoperative planning and discussion of the cases to be operated on, starting with the surgical anatomy and moving on to the finer points of the procedure, and that the case mix in the day's operation list should take all residents in different levels of training into account. There is a significant difference across consultants in terms of involvement in resident teaching in the elective and emergency surgeries during the duty times in particular. Regular evaluations of the current status of the programs by the authorities using such instruments and other techniques should be conducted and work to improve on the identified areas of weakness. Some of the things mentioned as strengths and that all consultants and senior residents should adopt are friendly communication between residents and the anesthesia, nursing staff, and consultants, proper preoperative discussion about the scheduled cases in some departments, supportive and encouraging instructional environments in the OT, and the provision of intraoperative feedback by consultants.

• “Some consultants focus only on finishing the operation early and don't want to give appropriate parts of the procedures even for senior residents and the OT is kept silent till the end of the operation and we feel like we are there only to assist them.”
• “Teaching must be according to the level of residency. Operation list, case mix, in a day should consider all levels of training.”
• “Some seniors are reluctant to share information for junior residents as the discussion is primarily with available senior residents.”
• “Few subspeciality case exposure especially among junior residents (urology, pediatric and plastic surgery.

## Discussion

The teaching and learning process is greatly influenced by the educational environment. OREEM can be used to determine the strengths and weaknesses of the operation theatre teaching in the surgical residency program. It has been used to assess the educational environment of the residents in different studies. For the first time since the programs' inception, we employed the OREEM measure as a tool to assess the OR educational environment of the postgraduate surgical residency programs at St. Paul's Hospital Millennium Medical College.

The average age of the surgical trainees was 28.5 + 2.1 years, making up a youthful population. This composition resembles that of other countries including Nigeria, the Netherlands, and the United States [24]. Our surgical training was notable for having a male gender predominance of 79.8%, which is low compared to the percentage of female medical school graduates. Similarly, up to 22% of surgical trainees in the United States and 28% of surgical trainees in the Netherlands were women [25,26]. More thorough qualitative research will be necessary to identify the precise obstacles.

Overall, the educational environment was found to be satisfactory based on the OREEM mean score of 69% in this study [27], even though it has been argued that any score below 80% in the nonparametric scale of the Likert scale is less than satisfactory [15]. A study conducted among Saudi Arabian urology residents revealed a similar total inventory score of 67.95% [28]. Another study from Nigeria that included 33 surgery residents reported one of the lowest scores of 69.74 [14]. On the other hand, higher scores have been reported from the studies done in the UK and Canada with OREEM scores of 79.16% and 74.4% respectively [29]. More favorable overall scores have been observed when comparing our results to research from other departments, such as pediatrics, obstetrics and gynecology, and intensive care training [30]. Local studies have also revealed that there is a concerning difference in how consulting surgeons and surgical residents see the instructional environment in the operating room [6].

Analysis of subscale scores showed the subscale of operating room “teaching and training” had the highest score with a score of 73.7% and the lowest scoring subscale was “Learning opportunities” at 62.7% which is reflected in 3 of the lowest scored items (no. s 27,19 and 20). Similarly, the original study conducted for the validation of the questionnaire among Scottish basic trainees indicated that the highest rated subscale was “teaching and training” and the lowest subscale score for the Scottish trainees was “learning opportunities” [27]. Other studies have also reported similarly positive scores for the “teaching and training” subscale. However, the “learning opportunities” subscale has been positively reported in other studies with mean scores of 72.6% and 76.21% [15,22].

A sizable number of residents expressed similar concerns in the free text and comments section indicating that there aren't many duty hours, there isn't adequate exposure to emergency cases, and that senior residents scrubbing on almost every case limits junior residents' exposure in the elective OR schedule. These and the acceptable but low score in the “learning opportunities” subscale suggest that the programs should seek to increase the exposure to “hands-on” experience in the operating room. Similar to this, an assessment of postgraduate medical education in sub-Saharan Africa that was published in 2019 cited the inadequate or limited opportunity to acquire procedural skills as an issue [31] and One of the areas identified for improvement in Neal Rupani's study on UK surgical residents was expanding learning opportunities [29].

The total OREEM score was further categorized by gender and it was discovered that there was no appreciable difference between the scores for females and males. However, comparing male and female responses in the OREEM subscale scores, female scores showed a statistically significant difference (*P* = 0.001) on the subscale of “atmosphere,” compared with males, as evidenced by corroborating item analysis. Statements no. 29 “The staff in the operating theatre is friendly. “The staff in the operating theatre is friendly,” no. 30 “I feel (not) discriminated against in theatre because of my sex,” no. 31 “I feel discriminated against in theatre because of my race,” all fall within the “learning opportunities” subscale and female residents gave them significantly lower ratings than their male counterparts. Other items also were rated less favorably by female respondents including statements no. 6 “My consultant's surgical skills are very good.”, no. 8 “My consultant (do not) immediately takes the instruments away when I do not perform well.”, and no. 9 “Before the operation my consultant discusses what part of the procedure I will perform.” Nagraj also had similar findings with no significant difference in the Overall score, between male and female responders, however, female residents perceived a much more positive operating theatre educational environment (score of 176) than did their male colleagues (score 153) [23]. Other studies did not indicate the presence of any difference in the total or any of the subscales based on gender [13,22]. The causes of this disparity were not determined in this study.

The overall score did not indicate any differences between junior and senior residents in their perception of the operating room educational environment similar to the studies conducted by kanashiro among Canadian general surgery residents and Ibrahim et.al [14,15]. However, on the subscale of “learning opportunities,” and “atmosphere” junior residents scored lower than senior residents; this difference was statistically significant (*p*-value < 0.05). There are mixed results regarding the difference in perceptions between senior and junior residents with some studies reporting no statistically significant differences regarding the total measure score or any of its subscales' scores [23,28] and others the reverse [22]. This could be explained by the fact that juniors typically observe and help more in the operating room, while seniors typically perform the majority of surgical procedures with assistance, have a more supervisory role in the ward, and carry out administrative tasks like scheduling. Junior residents also scored worse on five survey questions (p < 0.05), including those relating to opportunities afforded to trainees to ‘scrub in’ and practice their skills, the operative case-mix, and pressure placed on trainees to ‘hurry up. This has been similarly reflected in the comments section by several residents.

The Cronbach α was high at 0.865 for the 40 statements. This high reliability of the OREEM questionnaire is comparable to other studies [7,14,15] and supports the fact that the OREEM questionnaire has a high internal consistency when administered among various groups of residents in different environments. The Cronbach α also was high at 0.868 for the teaching-and-training domain, and it was good at 0.770 for “supervision/ workload/support” perception and at 0.684 for “learning opportunities” domains, which are comparable to those reported by others. Our results showed that the OREEM's content internal consistency is acceptable and valid to distinguish between various influences that contribute to the educational environment in the operating room and indicate that the separate items/subscales of this questionnaire appear to consistently measure the construct of the educational environment in the OR. When our data were analyzed to exclude each question in turn, using the “alpha if item deleted”, no significant improvement was seen with the removal of any of the 40 questions.

### Strengths and limitations

This study has some limitations that should be considered. First, the research was limited to surgical programs at a single center in Ethiopia's capital. This limits the applicability of the findings to all surgical residents and further studies including other available teaching institutions in different cities should be done. Second, this was a cross-sectional study, and causative inferences cannot be made. and further studies including other available teaching institutions in different cities should be done. Survey methodology is subject to potential response biases, in particular social desirability bias. The steps taken to protect respondent identity during data collection efforts were meant to reduce this bias. The study also has important strengths. This study included a larger number of surgical residents compared to previous studies and included residents from all years of training, representing different stages in the educational process and hence providing valuable information about the educational environment in the OR of surgical residency programs in Ethiopia. Furthermore, since this was the first study on the operating theatre educational environment in Ethiopia, this helps further underscore some of the components of OREEM and showed it to be a reliable instrument.

## Conclusion and recommendations

The overall OREEM score was found to be at a satisfactory level of 69% (138/200), indicating that the residents had good perceptions of their training and supervisors, learning opportunities in the operation theatre, the atmosphere in the operation theatre, and supervision being provided to them. The highest score was for the operating room “teaching and training” and the lowest score was for the operating room “Learning opportunities” subscales. In addition, we noted that gender affected the perception of the residents about the OR learning environment mainly regarding the ‘atmosphere” scale whereas the level of training affected the “learning opportunities” and “atmosphere” domains.

Creating an optimal learning environment in the operating room is pivotal for the technical proficiency of surgeons. The study suggests collaborative efforts among residents, academic personnel, and college administrators. Recommendations include increasing residents' duty hours, diversifying case exposure, and enhancing preoperative planning and discussion. Emphasizing sustained interactive intraoperative engagement, coupled with constructive feedback, is crucial, necessitating faculty development sessions. Addressing the gender-based gap in the perception of the teaching atmosphere calls for proactive measures to understand and address concerns expressed by female residents within surgical residency programs.

## Funding

St. Paul Hospital Millennium Medical College funded the study but had no involvement from the study design to submission of the paper for publication.

## Ethical approval

Ethical clearance was obtained from SPHMMC IRB.

## CRediT authorship contribution statement

**Goytom Knfe:** Conceptualization, Data curation, Formal analysis, Funding acquisition, Investigation, Methodology, Project administration, Resources, Software, Writing – original draft, Writing – review & editing. **Henok Teshome:** Conceptualization, Methodology, Supervision, Validation, Writing – review & editing. **Maru Gama:** Methodology, Supervision, Validation, Writing – review & editing. **Engida Abebe:** Methodology, Supervision, Validation, Writing – review & editing. **Mulugeta Kassahun:** Methodology, Supervision, Validation, Writing – review & editing. **Berhanetsehay Tekelwold:** Conceptualization, Supervision, Validation, Writing – review & editing.

## Declaration of competing interest

There is nothing to declare.
